# *In vivo* Investigations of the Effect of Short- and Long-Term Recombinant Growth Hormone Treatment on DNA-Methylation in Humans

**DOI:** 10.1371/journal.pone.0120463

**Published:** 2015-03-18

**Authors:** Julia Kolarova, Ole Ammerpohl, Jana Gutwein, Maik Welzel, Inka Baus, Felix G. Riepe, Thomas Eggermann, Almuth Caliebe, Paul-Martin Holterhus, Reiner Siebert, Susanne Bens

**Affiliations:** 1 Institute of Human Genetics, Christian-Albrechts-University Kiel & University Hospital Schleswig-Holstein, Campus Kiel, Kiel, Germany; 2 Division of Pediatric Endocrinology and Diabetes, Department of Pediatrics, Christian-Albrechts-University Kiel & University Hospital Schleswig-Holstein, Campus Kiel, Kiel, Germany; 3 Institute of Human Genetics, University Hospital Aachen, Aachen, Germany; RWTH Aachen University Medical School, GERMANY

## Abstract

Treatment with recombinant human growth hormone (rhGH) has been consistently reported to induce transcriptional changes in various human tissues including peripheral blood. For other hormones it has been shown that the induction of such transcriptional effects is conferred or at least accompanied by DNA-methylation changes. To analyse effects of short term rhGH treatment on the DNA-methylome we investigated a total of 24 patients at baseline and after 4-day rhGH stimulation. We performed array-based DNA-methylation profiling of paired peripheral blood mononuclear cell samples followed by targeted validation using bisulfite pyrosequencing. Unsupervised analysis of DNA-methylation in this short-term treated cohort revealed clustering according to individuals rather than treatment. Supervised analysis identified 239 CpGs as significantly differentially methylated between baseline and rhGH-stimulated samples (p<0.0001, unadjusted paired t-test), which nevertheless did not retain significance after adjustment for multiple testing. An individualized evaluation strategy led to the identification of 2350 CpG and 3 CpH sites showing methylation differences of at least 10% in more than 2 of the 24 analyzed sample pairs. To investigate the long term effects of rhGH treatment on the DNA-methylome, we analyzed peripheral blood cells from an independent cohort of 36 rhGH treated children born small for gestational age (SGA) as compared to 18 untreated controls. Median treatment interval was 33 months. In line with the groupwise comparison in the short-term treated cohort no differentially methylated targets reached the level of significance in the long-term treated cohort. We identified marked intra-individual responses of DNA-methylation to short-term rhGH treatment. These responses seem to be predominately associated with immunologic functions and show considerable inter-individual heterogeneity. The latter is likely the cause for the lack of a rhGH induced homogeneous DNA-methylation signature after short- and long-term treatment, which nevertheless is well in line with generally assumed safety of rhGH treatment.

## Introduction

Recombinant human growth hormone (rhGH) has been introduced in 1985 for the treatment of children with short stature [1]. Current indications include among others growth hormone (GH) deficiency, children born small for gestational age (SGA), Turner syndrome, Prader–Willi syndrome, chronic renal insufficiency and short stature homeobox (*SHOX*) gene deficiency [2]. While some authors reported rhGH treatment to be associated with adverse side effects like increased risk for leukemia or secondary neoplasms [3,4,5,6,7], this has not been confirmed by others [8,9,10].

Physiologically, GH is secreted from pituitary somatotrophic cells. It regulates postnatal growth through a complex network of direct and indirect effects involving amongst others the stimulation of insulin-like growth factor 1 (IGF1) synthesis and release in the liver [11]. Analogous to the physiologic GH effect, treatment with rhGH enhances circulating IGF1 levels. The rise of IGF1 concentration in the peripheral blood following rhGH treatment (so called IGF1 generation test, IGFGT) serves, thus, in the clinical setting as basic laboratory parameter to estimate the response to rhGH treatment *in vivo* [12].

In order to identify *in vivo* targets of the GH-axis and eventually develop better prediction models to monitor response to rhGH therapy a series of microarray-based gene expression studies has been conducted [13,14,15]. Indeed, albeit variable in design and of restricted power due to small sample sizes, transcriptional changes following rhGH treatment were consistently observed in peripheral blood mononuclear cells (PBMCs).

Determination of gene expression patterns involves epigenetic modifications, including DNA-methylation [16,17]. For hormones other than GH it has been shown that transcriptional effects induced by these hormones are conferred or at least accompanied by DNA-methylation changes [18,19,20]. Altered DNA-methylation is also a feature of many diseases, including diabetes and cancer [21,22]. Thus, the characterisation of epigenetic modifications induced by rhGH treatment might not only contribute to the understanding of the GH response but could also provide clues for potential adverse effects of rhGH treatment.

To analyse effects of rhGH treatment on the DNA-methylome, we performed an array-based analysis of DNA-methylation in two independent cohorts of 24 and 36 children treated short-term and long-term with rhGH, respectively.

## Material and Methods

### Ethics Statement

The study has been approved by the Ethics Committee of the Medical Faculty of the Christian-Albrechts-University Kiel (AZ B305/08, D401/08, Amendment 06/03/2012 and AZ D422/07). All patients and in minors their caretakers gave written informed consent upon inclusion into the study.

### Study population for short-term effects of rhGH on DNA-methylation

**Fig 1:**
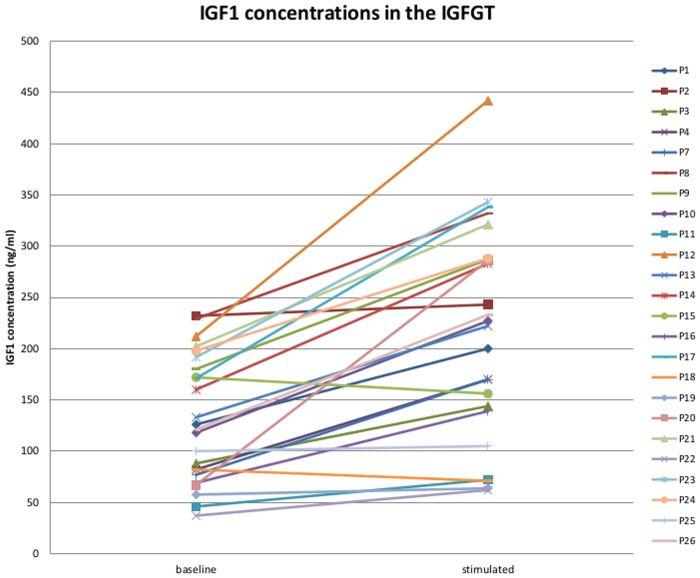
Increase of IGF1 concentration upon rhGH treatment in the IGFGT. Scatter plot of the IGF1 concentrations (ng/ml) at baseline and rhGH stimulated blood sampling among the short-term rhGH treatment study cohort. The two measurements per sample are connected. Sample numbering corresponds to the sample identifier in Table 1. A significant increase in IGF1 concentrations after rhGH treatment is observed (paired t-test, p = 1.93 x 10–6).

PBMC samples from 24 patients were included upon initialisation of rhGH treatment. Diagnoses leading to rhGH treatment in this cohort were classical GH deficiency (n = 7, one with panhypopituitarism, one also born SGA), neurosecretory dysfunction leading to GH deficiency (n = 6), SGA with lack of catch-up growth (n = 7), qualitative GH deficiency (Kowarski syndrome (n = 2)), Turner syndrome (n = 1), primary IGF1 deficiency (n = 1). Median age of the patients in this cohort was 9.17 years (range: 5.17–15.42 years). For details on patients’ characteristics see Table 1. As part of routine clinical work-up all patients underwent an IGFGT. The first blood sample (baseline) was drawn prior to the first dose of rhGH and the second blood sample (stimulated) in the first morning between 8 and 10 a.m. after 4 days of rhGH treatment (30 μg/kg body weight/day). The actual lower leg length was recorded with a knemometer according to the method extensively described by Hermanussen et al. in [23] upon baseline and stimulated blood sampling and the knemometry rate over the lower limbs was calculated from both measurements (Table 1). IGF1 and IGFBP3 concentrations (RIA, Mediagnost, Reutlingen, Germany) were determined upon baseline and stimulated blood sampling (Table 1, Fig. 1).

**Table 1:** Clinical characterisation of the 24 children investigated for short-term effects of rhGH on DNA-methylation.

Sample ID	Age (years)	Diagnosis	Sex	IGF1	IGFBP3 baseline in μg/ml (centile)	IGF1 stimulated in ng/ml	IGFBP3 stimulated in μg/ml	Knemometry rate (mm)
				baseline in ng/ml (centile)				
P1	13.00	NSD	m	126 (2)	2.23 (6)	200	3.49	0.1
P2	11.58	SGA	f	232 (24)	3.29 (45)	243	3.4	0.75
P3	13.33	NSD	m	88 (<0.1)	1.97 (1)	144	2.98	0.4
P4	8.92	STH-D~	f	82 (2)	2.37 (27)	170	2.74	-0.1
P7	7.92	Q-STH-D	m	77 (4)	1.22 (<0.1)	170	1.38	0.5
P8	7.67	SGA	m	229 (86)	3.5 (93)	332	2.1	4.1
P9	12.33	Q-STH-D	m	180 (18)	2.43 (12)	287	3.53	0.9
P10	10.67	NSD	m	118 (5)	1.8 (2)	227	2.47	1
P11	7.33	STH-D	m	46 (0.4)	1.58 (2.6)	72	2.53	0.55
P12	9.42	SGA	m	212 (76)	3.95 (94)	442	4.39	1.3
P13	8.67	NSD	f	133 (18)	2.34 (25)	222	2.78	0.2
P14	15.42	STH-D	m	160 (0.2)	3.27 (42.1)	283	3.87	1.1
P15	12.92	NSD	f	172 (2.2)	3.14 (37.9)	156	3.71	0
P16	6.00	SGA	m	69 (16.5)	2.66 (64.1)	139	2.85	1.2
P17	11.58	NSD	f	171 (9.6)	3.35 (53.3)	338	3.5	0.75
P18	6.42	IGF1-D	f	82 (22.3)	1.75 (7.7)	71	1.67	0.3
P19	6.17	SGA	m	58 (9)	1.14 (0.1)	64	2.82	-0.5
P20	13.33	STH-D, PAN	m	67 (0)	1.6 (0.1)	286	4.39	0.6
P21	11.75	STH-D	f	202 (16.5)	3.03 (34.4)	321	3.14	0
P22	5.17	SGA	m	37 (1.1)	1.69 (9.8)	62	2.44	0.3
P23	6.92	SGA, TS	f	192 (85.5)	1.85 (11.6)	343	4.66	1.1
P24	13.17	STH-D	m	198 (29.4)	2.77 (27.2)	288	1.89	0.5
P25	8.00	STH-D, SGA	f	100 (15.2)	2.76 (52.5)	105	2.44	0.2
P26	5.83	UTS	f	121 (59.3)	3.02 (83)	233	4.04	0.1

NSD: GH deficiency due to neurosecretory dysfunction; SGA: small for gestational age; STH-D: classical GH deficiency; Q-STH-D: qualitative GH deficiency (Kowarski syndrome); IGF1-D: IGF1 deficiency; PAN: panhypopituitarism; TS: Temple-Syndrome (UPD(14)mat syndrome); UTS: Turner-Syndrome. ~: STH-D following pontine tumor. IGF1 and IGFBP3 concentration measured at collection of baseline blood sample. Knemometry rate measured according to [23]. m: male; f: female.

### Study population for long-term effects of rhGH on DNA-methylation

Peripheral blood (PB) samples from 54 children born SGA, defined as birth length and/or birth weight -2SD below the mean for gestational age according to the reference data published by Niklasson et al. [24], were included. The cohort has been recruited in the framework of the consortium “Diseases caused by imprinting defects: clinical spectrum and pathogenetic mechanisms” and partially overlaps with the previously described cohort of SGA born children studied for their DNA-methylation pattern at 10 imprinted loci by bisulfite pyrosequencing [25]. Of note, the children with evidence for DNA-methylation defects at the loci *IGF2*, *H19*, *GRB10*, *MEG3* and *NDN* in the latter study were excluded from the present study. Out of 54 children, 36 received treatment with rhGH (median therapy interval at inclusion: 33 months, interquartile range: 15.5–62.25 months) and 18 not. Clinical parameters were obtained by standardised questionnaires and are summarized in Table 2.

**Table 2:** Clinical characterisation of the 54 children born SGA investigated for long-term effects of rhGH treatment on DNA-methylation.

Criteria	Children treated with rhGH	No data	Children without rhGH treatment	No data
number of included children	36	0	18	0
female	8	0	5	0
male	28	0	13	0
rhGH treatment in months (median and IQR)	33 [15.5–62.25]	0	0	0
developmental delay	6	3	4	0
**Prenatal factors**				
maternal nicotin consumption	8	5	4	1
EPH gestosis	2	8	2	3
placenta insufficiency	9	14	5	4
**Parametric data (median and IQR)**				
age at investigation (in years)	10.63 [7.75–12.65]	0	5.67 [4.33–9.23]	0
birth weight (SDS)	-3.29 [-2.59–-4.25]	0	-2.88 [-2.43–-3.76]	0
birth length (SDS)	-2.54 [-2.08–-3.42]	0	-2.5 [-2.22–-3.37]	0

rhGH: recombinant human growth hormone; EPH gestosis: edema-proteinuria-hypertension gestosis; IQR: interquartile range; SDS: standard deviation score.

### PBMC isolation, DNA extraction and bisulfite conversion

For the short-term rhGH treatment cohort PBMC isolation from freshly drawn blood samples was performed according to [15]. Briefly, PBMC were isolated by Ficoll (Biochrom AG, Berlin, Germany) gradient centrifugation, resuspended in RPMI 1640 medium (Gibco, Karlsruhe, Germany) with 10% DMSO, 10% heat-inactivated FCS, penicillin (100 U/mL), streptomycin (100 μg/mL), and L-glutamine (2 mM) and frozen in liquid nitrogen.

DNA was extracted from PBMC (short-term cohort) or PB (long-term cohort) using standard methods and 1 μg DNA was bisulfite converted using the EZ DNA Methylation Kit (Zymo Research, Orange, CA, USA) according to the manufacturer's instructions.

### Array-based DNA-methylation analysis

For DNA-methylation analysis the Infinium HumanMethylation450 BeadChip (Illumina, Inc., San Diego, CA, USA) was used according to the manufacturer’s instruction. This platform allows the interrogation of 485,577 assays (482,421 CpG sites, 3091 non-CpG sites, 65 random SNPs) in parallel at a single-nucleotide resolution per sample [26]. Arrays were scanned using the Illumina iScan. Raw hybridisation signals were processed using the GenomeStudio software (version 2011.1; Methylation Analysis Module version 1.9.0, Illumina) applying the default settings and internal controls for normalisation. Quality criteria referring to the technical results of hybridisation were set as a gene call rate above 98% per sample and a detection p-value <0.01 per CpG site. After quality filtering a total of 48 hybridisations and 482,000 targets entered the final analyses in the short-term rhGH treatment cohort. In the long-term rhGH treatment cohort CpGs were additionally filtered for those targets corresponding to autosomes and those labeled as SNPs. This led to a total of 54 samples and 471,808 targets entering final analyses. Results are available in a MIAMI compliant format from Gene Expression Omnibus (GSE57205).

### Analyses of DNA-methylation data

For unsupervised and groupwise comparisons the Omics Explorer (ver.3.0 (25); Qlucore, Lund, Sweden) was used. For unsupervised analysis false discovery rate (FDR) and/or variance (σ/σ_max_) filters applied for the different analyses are given in the respective descriptions of the results. Groupwise comparisons were performed as supervised analysis applying t-test statistics.

In the short-term rhGH treatment group in addition a paired t-testing of avg.beta values was performed, both unadjusted and adjusted for multiple testing. For targets with a p-value <0.0001 in the paired t-test avg.beta values of baseline and stimulated samples were subjected to a cluster analysis (R version 3.0.1 and package ‘gplots’ function heatmap.2).

Moreover, in the short-term cohort delta beta values (avg.beta value stimulated sample subtracted from avg.beta value baseline sample) were calculated for all 482,000 targets entering final analysis. Delta beta values >0.1 or <-0.1 (corresponding roughly to a difference of DNA-methylation between both samples of a pair of +/-10%) were classified as differentially methylated. The cut-off of 10% was arbitrarily chosen to enrich for differentially methylated CpGs with likely biological significance [17]. Those targets in which at least 3 of the 24 sample pairs (>10%) show delta beta values of >0.1 or <-0.1 were subsequently subjected to cluster analysis as described above. Clustering was performed using the delta beta values as continuous variables as well as by classifying the targets as gained methylation (labeled 1 for delta beta values >0.1), lost methylation (labeled -1 for delta beta values <-0.1) and unchanged (labeled 0 for delta beta values <0.1->-0.1) in response to rhGH treatment.

Information about the location of differentially methylated CpG loci in regulatory regions in the lymphoblastoid cell line GM12878 given in S2 Table and S5 Table were available from Ensembl (ftp://ftp.ensembl.org/pub/release-78/regulation/homo_sapiens/RegulatoryFeatures_GM12878.gff.gz).

### Gene ontology analysis

Gene ontology analysis for enrichment of biological processes, function and protein binding among differentially methylated genes was performed with the Gene Annotation Tool to Help Explain Relationships GATHER, http://gather.genome.duke.edu/, accessed 05/06/2014 [27].

### Validation of DNA-methylation differences by bisulfite pyrosequencing

Based on delta beta values in the rhGH short-term cohort 5 loci (*GNLY*, *TRIM39*, *SLC15A4*, *IGF1R*, *SLC6A16*) were selected for validation. Selection criteria comprised at least three differentially methylated CpGs per locus in at least one, preferably multiple patient samples. Bisulfite pyrosequencing assays were designed to cover two of these differentially methylated CpGs per locus. Bisulfite pyrosequencing assays were designed using the PyroMark Assay Design Software (Version 2.0, Qiagen, Hilden Germany). For PCR and primer conditions as well as analysed sample pairs from the short term cohort see S1 Table. We used 1 μl of bisulfite converted DNA from PBMC as template for each PCR in a final volume of 25μl. The correct size of the PCR product was verified by gel electrophoresis. For purification of PCR products and single strand preparation a mastermix of 5μl streptavidin sepharose beads and 40μl binding buffer was prepared. 43μl of this mastermix was pipetted to the PCR products. Purification and single strand preparation of the PCR product were performed with the Vacuum Prep Tool (Biotage), followed by denaturation at 85°C for 2 minutes and sequencing primer hybridisation. Pyrosequencing was performed using the PyroMark ID and evaluated using the PyroMark CpG Software 1.0.11 (Biotage). Assays were validated using *in vitro* methylated DNA as positive control (Millipore, Hilden, Germany) and whole genome amplified DNA as unmethylated control.

## Results

In order to investigate the short-term and long-term influence of rhGH on DNA-methylation we analysed DNA-methylation patterns in two independent cohorts using the HumanMethylation450 BeadChip [26]. We will first separately report the results obtained in the short-term and long-term cohorts, respectively, and then provide data on the intersection of the results between both cohorts.

### Analyses of the short-term study cohort

#### Evaluation of biological response to rhGH treatment

The short-term study cohort comprised paired PBMC samples from 24 patients obtained during the initialisation of rhGH treatment (Table 1). There was a significant increase of the IGF1 concentrations after 4 days of rhGH treatment in this cohort (paired t-test p-value = 1.93 x 10^-6^) pointing to a significant biological response. On an individual level, an increase of IGF1 concentration was observed in 22/24 patients (Fig. 1). In one of the two cases without IGF1 rise the knemometry rate points to biological response.

#### Comparison of DNA-methylation of baseline and rhGH stimulated PBMC samples

**Fig 2:**
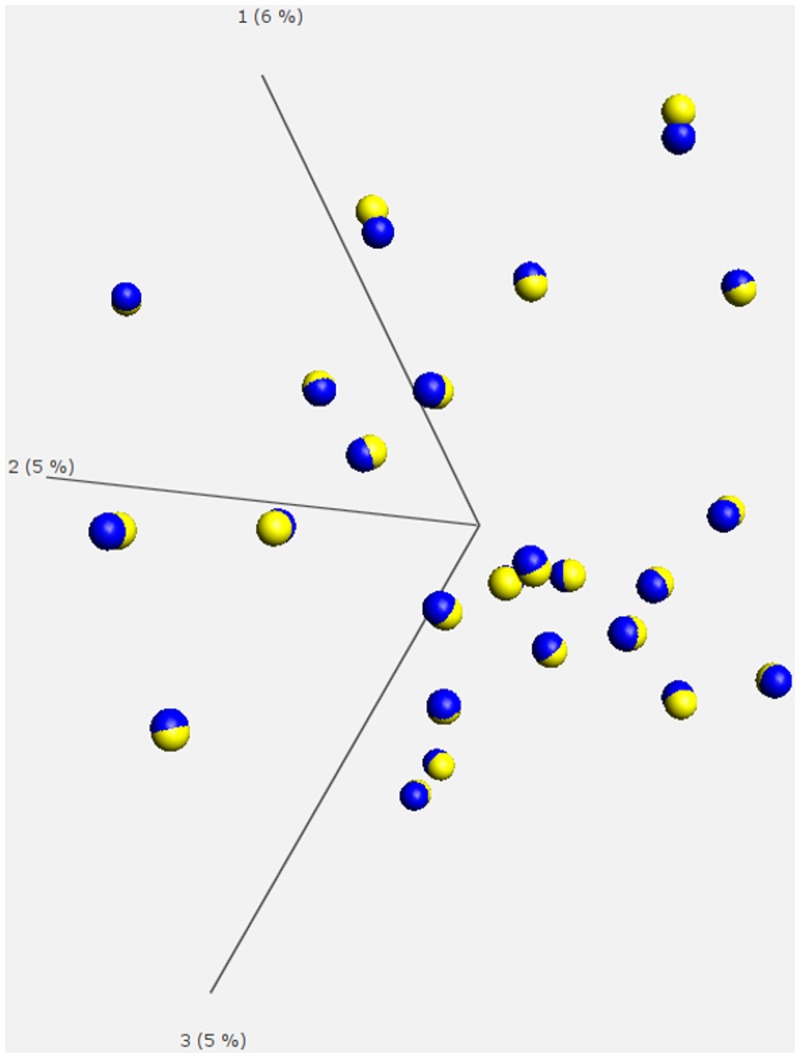
Principal component analysis (PCA) in the overall short-term rhGH treatment study cohort. Results of the PCA analysis performed by using a set of 4262 loci with the highest variance (filter of variance of σ/σ_max_>0.275) does not segregate the cohort into treatment groups. Instead, each sample pair showed close relationship to one another thereby proving that the individual origin of the samples has much stronger influence on DNA-methylation than rhGH treatment. rhGH stimulated samples are depicted in blue, baseline samples are depicted in yellow.

To investigate the short-term effects of rhGH on *in vivo* DNA-methylation we first globally compared the DNA-methylation values of all baseline and stimulated PBMC samples in the short-term cohort. A principal component analysis (PCA) of the complete short-term data set revealed no significant separation in different subgroups. PCA analysis using the 4262 loci with the highest variance (filter of variance σ/ σ_max_>0.275) grouped the sample pairs of each patient but failed to segregate the cohort into treatment groups thereby proving that the individual origin of the samples has much stronger influence on DNA-methylation than rhGH treatment (Fig. 2). An unpaired supervised comparison according to rhGH treatment revealed none of the evaluated CpG loci to be significantly (FDR<0.05) differentially methylated between baseline and stimulated samples. Nevertheless, a paired t-test of the 482,000 targets passing quality filtering identified 239 CpGs as significantly differentially methylated (p < 0.0001) among baseline and stimulated samples (S2 Table). The absolute differences of the mean DNA-methylation between baseline and stimulated samples for the significantly differentially methylated loci reached a maximum of 0.041 (corresponding roughly to a difference of DNA-Methylation of 4.1%). This maximum was determined for the CpG site cg11283402 associated with the gene *ARID5B*. Gene ontology analysis of the 162 genes associated with the 239 CpG loci revealed significant enrichment among the annotation category “protein binding” for e.g. BCL6 (B-cell CLL/lymphoma 6), HDAC4 (histone deacetylase 4) and HDAC7A (histone deacetylase 7A) (S3 Table and S4 Table). However, in line with the results of the unsupervised and unpaired supervised analysis, none of the 239 CpG loci remained significantly differentially methylated any more after adjustment for multiple comparisons at a FDR of 0.05.

**Fig 3:**
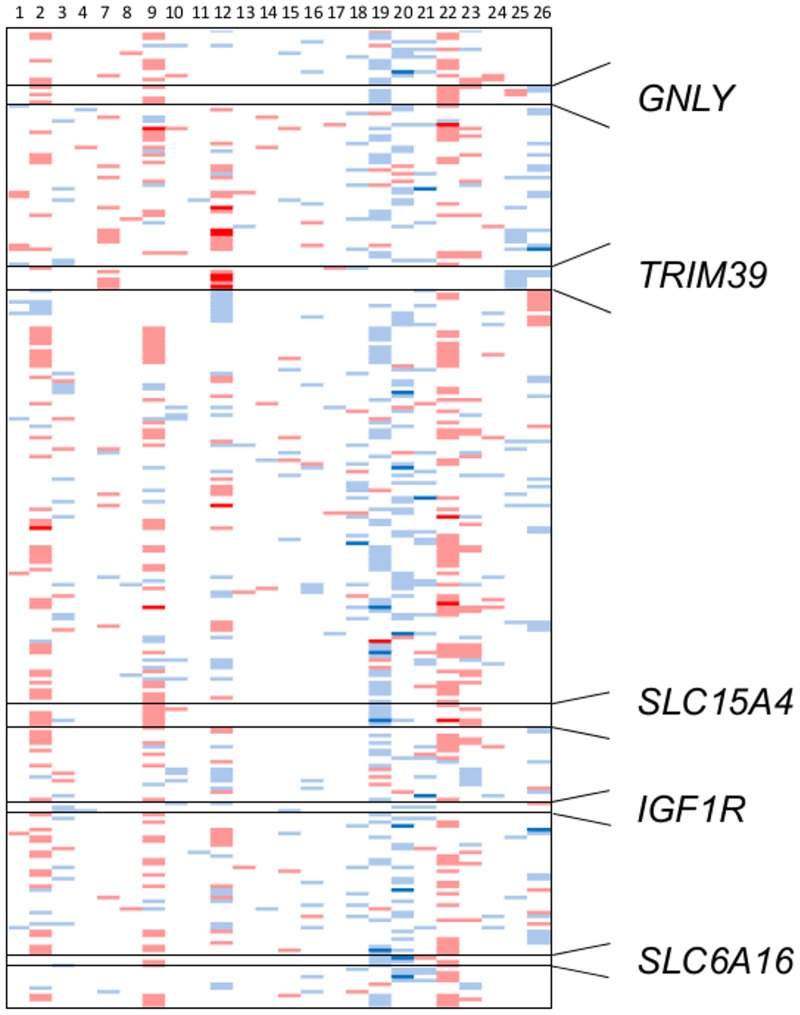
DNA-methylation results in the short-term rhGH treatment study cohort using an individualised evaluation approach. Differences of avg.beta values between stimulated and baseline samples (delta beta) of each pair were calculated. Depicted are those loci, in which at least 3/24 analysed sample pairs showed absolute delta beta values below -0.1 or above 0.1 (roughly corresponding to a difference of DNA-methylation of +/- 10% between each analysed sample pair) and in which at least 3 CpGs were affected per gene (n = 259). Red: delta beta values above 0.2, pale red: delta beta values between 0.1 and 0.2. Blue: delta beta values below -0.2, pale blue: delta beta values between -0.1 and -0.2. Samples are sorted according to Table 1 with P1 at the left margin and P26 at the right margin. Those loci used for bisulfite pyrosequencing validation are zoomed out and shown at the right side of the figure.

Because both, the diagnosis leading to rhGH treatment and the response to rhGH—measured by IGF1 and IGFBP3 increases in plasma as well as by increases of knemometry rates—showed marked inter-individual heterogeneity we additionally aimed at a more individualised evaluation of the DNA-methylation changes. Thus, the difference of avg.beta values between stimulated and baseline samples (delta beta) of each pair was calculated. A total of 2350 CpG and 3 CpH loci showed absolute differences between baseline and stimulation of at least 0.1 in at least 3 of the 24 pairs (Fig. 3, S5 Table). Gene ontology analysis of the 1426 genes associated with these 2353 loci revealed among others significant enrichment for the processes “cell communication”, “immune response” and”signal transduction” (S6 Table and S7 Table).

Comparison of the 162 and 1426 genes identified with the unadjusted paired t-test and the individualised approach revealed 23 overlapping genes (S8 Table). Those 23 genes showed significant enrichment for the processes “imprinting”, “DNA methylation” and “DNA alkylation” as well as protein binding to “BCL6” and “HDAC1, HDAC4, HDAC6” (S9 Table and S10 Table). Neither cluster analysis of the 239 CpGs detected by the unadjusted paired t-test nor of the 2353 loci detected with the individualised approach as differentially methylated between baseline and stimulated samples showed grouping according to the underlying diagnosis.

#### Validation of DNA-methylation differences by bisulfite pyrosequencing

Differences observed by the array-based analyses were validated by bisulfite pyrosequencing for 5 loci selected based on delta beta values (*GNLY*, *TRIM39*, *SLC15A4*, *IGF1R*, *SLC6A16*) in the short-term cohort. The bisulfite pyrosequencing assays interrogated for each selected locus two CpGs previously investigated on the array. For each of the 10 investigated CpGs 1 to 6 sample pairs of the short-term cohort were analysed, resulting in a total of 34 paired measurements (see S1 Table for details). A comparison of the results obtained by both techniques at the investigated CpGs showed high correlation (Pearson r = 0.78, p-value = 4.015 x 10^-8^; S1 Fig).

### Analyses of the long-term study cohort

#### Comparison of DNA-methylation between rhGH treated and untreated peripheral blood samples

Complementary to the investigations in the short-term cohort we analysed an independent cohort of children born SGA for long-term effects of rhGH treatment. This cohort consisted of peripheral blood (PB) samples from 54 children born SGA, among which 36 received rhGH treatment for a median of 33 months (interquartile range 15.5–62.25 months) and 18 received no rhGH treatment (Table 2). Importantly, no paired pre-treatment and follow-up samples were available from the rhGH treated patients in this cohort. Thus, and because the long-term comparison in a longitudinal study could be confounded by age-related DNA methylation changes, the statistical approach differed from the short-term cohort and we compared the rhGH treated patients to an independent cohort of untreated patients matched with regard to SGA (see methods section and Table 2). All autosomal target loci fulfilling quality filtering (n = 471,808) were subjected to the analysis. Nevertheless, neither unsupervised nor supervised analyses (FDR<0.05) revealed a separation of the overall cohort into distinct subgroups or lead to the identification of significantly differentially methylated CpGs between rhGH treated and untreated individuals. A linear regression analysis of DNA-methylation results and period of rhGH treatment revealed no correlation for any of the CpG sites analysed. Thus, in this cohort we could not identify a homogeneous effect of long-term rhGH treatment on DNA-methylation in the peripheral blood.

#### Analysis of the long-term cohort for loci identified as differentially methylated in the short term cohort

Finally, we investigated the long-term cohort only for those targets detected as differentially methylated in the short-term rhGH treatment cohort. A total of 235 of the 239 CpGs and 2280 of the 2353 targets, respectively, identified in the short-term cohort matched the (quality) filter criteria in the long-term cohort. Of those none was differentially methylated between the treatment groups in the long-term cohort (supervised analysis, FDR<0.05). Similarly, there was no significant correlation of the methylation levels of those targets and the treatment interval.

## Discussion

In this study we aimed at characterising short- and long-term effects of rhGH treatment on the DNA-methylome. We identified marked intra-individual responses of the DNA-methylation to rhGH treatment, primarily when comparing paired samples in the short-term treatment setting. These, however, seem to be inter-individually heterogeneous.

### Groupwise DNA-methylation changes associated with rhGH treatment

During the initial phase of rhGH treatment 162 genes were detected as significantly differentially methylated by paired unadjusted t-testing in the short-term treatment cohort. Despite none of these genes retained significance after correction for multiple testing, we investigated whether these genes represent putative candidate genes to eventually integrate in a (epi)genetic prediction model for rhGH response and whether these targets are persistently affected during long-term rhGH treatment. Nevertheless, by analysing paired samples in a longitudinal approach over a long period of time (i.e. several years) and during a critical phase in development (i.e. childhood and adolescence) it needs to be taken into account that the effects of rhGH treatment on the IGF1 signaling pathway are inevitably influenced by the effects of ageing [28,29,30]. Indeed, it is well known that ageing affects DNA methylation in peripheral blood [29,30]. We therefore had to adapt the study design in the long-term treatment cohort. Though our approach takes aging into account, it lacks power as compared to a paired design and is less controlled for diagnosis heterogeneity. We therefore chose for the long-term analysis a homogeneous cohort of probands, which uniformly received rhGH treatment due to the condition SGA. Principal component analysis indeed indicated the long-term cohort to represent a homogeneous study batch. Neither unsupervised nor supervised analysis regarding rhGH treatment among all CpGs after quality filtering and in the subset of CpGs detected as differentially methylated by unadjusted paired t-testing in the short-term cohort revealed a separation among the long-term cohort. These findings uniformly point to a lack of a rhGH induced homogeneous DNA-methylation signature after rhGH treatment, which is well in line with the generally assumed safety of rhGH treatment.

### Individual DNA-methylation changes associated with rhGH treatment

Indications to start rhGH treatment are numerous, pointing to high variability of the individual environment in which the rhGH exerts its effect. To address this point we performed unsupervised analyses with the various data sets. Whereas these analyses did not identify considerable subgroups of patients they clearly showed the closest relation of DNA-methylation patterns between both samples of the same individual in the short-term cohort (Fig. 2). These findings prompted us to take advantage of the paired study design in the short-term cohort and compare the sample pairs in an individualised approach leading to the detection of 1426 recurrently differentially methylated genes in at least 3/24 pairs. Exemplary validation for the targets detected as recurrently differentially methylated in the individualised approach showed good correlation suggesting that these differences are technically—and likely also biologically—robust. The direction of identified and validated DNA-methylation changes in response to rhGH stimulation is frequently heterogeneous, which is in concordance to the observed effects of androgens on the DNA methylome [18,19].

### rhGH and immune system

We cannot reliably distinguish whether the DNA-methylation changes observed in the individualised approach are attributable to selective *de novo* DNA-(de)methylation, to more general effects like individual host factors or to expansion of a distinct cell population. Nevertheless, gene ontology analyses of these genes indicate that a subset of patients shows a response of immune cells in the initial phase of rhGH treatment. Of note previous studies showed no changes in the number of T- and B-lymphocytes upon short-term *in vitro* incubation with growth hormone releasing hormone [31], while the number of B-lymphocytes in long-term rhGH treated children was reported to decrease [32,33,34]. Approximately 90% of B-lymphocytes and monocytes express GH receptors on their cell surfaces, while they were variably detected on T-lymphocytes and natural killer cells [35,36]. Thus, rhGH has the potential to regulate all major immune cell types and influence immune function [37]. The association of rhGH treatment and the immune system we observed in our study corroborates several previous studies on gene expression [13,14,15]. Welzel et al. performed gene expression studies in PBMC in the frame of the IGFGT and detected inter-individual variability in rhGH-related response. Nevertheless, gene ontology analyses revealed significant over-representation of T-cell and B-cell activation, Toll receptor and JAK/STAT signaling pathways among the 313 transcripts associated with GH treatment [15]. Of those 20 transcripts overlap with the differentially methylated loci identified herein. Among them is *BCL6* encoding a master regulator of B- and T-cell development [38]. The implication of the JAK/STAT signaling pathway in GH treatment has been further proven by Trovato et al. [39]. By analysing GH gene expression changes in PBMC of 10 adult GH deficient women after 4 weeks of GH treatment Fernández-Pérez et al. proposed 24 candidate genes to discriminate between samples before and after GH treatment, which also include genes involved in immune response and signal transduction [14]. Whatmore and colleagues studied gene expression changes in PBMC after 3 months of rhGH treatment [13]. Gene ontology analysis of one of the cluster patterns associated with GH treatment, referring to 179 probes, again included immune function genes [13]. Thus, aside from a recurrent theme also in the gene expression studies pointing to considerable individual differences there is compelling evidence that (rh)GH treatment affects expression of immune-related genes either directly or through regulating growth of certain immune cell populations.

## Conclusions

In summary we observed strong inter-individual differences of DNA-methylation upon rhGH treatment in the short-term treatment cohort, primarily related to a response of immune cells. These might be caused by a direct effect of rhGH on DNA-methylation or by growth differences in cell populations with differential rhGH sensitivity in the peripheral blood. The observed heterogeneity of DNA-methylation changes might be influenced by the host variability, e.g. individual differences in GH receptor and IGF1 receptor expression or the complex polygenic background on which GH effects are exerted. We did not observe the establishment of a rhGH induced homogeneous DNA-methylation signature after short- and long-term treatment, thereby corroborating the generally assumed safety of rhGH treatment. However, these results cannot preclude uniform DNA-methylation changes in rhGH target tissues, like liver and cartilage, tissues hardly available in a clinical setting based on ethical considerations. Moreover, DNA methylation changes are generally considered to represent rather long-term epigenetic modifications that may not be affected by a short-term rhGH treatment. Therefore, in a future approach, it would be conceivable to investigate if short-term treatment has an impact on more dynamic (short-term) epigenetic modifications such as histone methylation and acetylation by analyzing genome-wide changes in histone modifications by ChIP-Seq in the studied short-term cohort.

## Supporting Information

S1 Fig Comparison of the results obtained by the array-based approach and bisulfite pyrosequencing in the short-term rhGH treatment study cohort. Bisulfite pyrosequencing assays were designed for 5 loci (*GNLY*, *TRIM39*, *SLC15A4*, *IGF1R*, *SLC6A16*). Within each locus 2 CpGs covered by the array-based approach were evaluated. For each of the 10 investigated CpGs 1 to 6 sample pairs were analysed, resulting in a total of 34 paired measurements. **A: Correlation analysis.** Depicted are the delta beta values (value of the stimulated samples subtracted from the value of the baseline sample) for the 34 paired measurements. A comparison of the results obtained by both techniques at the investigated CpGs showed high correlation (Pearson r = 0.78, p-value = 4.015 x 10^-8^). **B: Bland-Altman plot.** Bland-Altman plot of DNA-methylation results from all 68 single measurements obtained by bisulfite pyrosequencing and the corresponding avg.beta values obtained from the array-based measurements. For this figure avg.beta-values as well as methylation levels determined by bisulfite pyrosequencing are displayed as percent values in order to obtain data sets of the same size range. The difference between both methods for every individual measurement is plotted against the average of both methods. The average of the differences +/− two times the standard deviation denotes the 95% range for the limits of agreement (marked by the dotted horizontal lines).
(TIF)

S1 Table Primers and PCR conditions used for bisulfite pyrosequencing assays. (XLSX)

S2 Table Lists of targets identified as differentially methylated among the rhGH short-term study cohort by paired t-test. (XLSX)

S3 Table Gene ontology analysis among the targets listed in S2 Table. Analysis was performed with the Gene Annotation Tool to Help Explain Relationships GATHER, http://gather.genome.duke.edu/, accessed 05/06/2014 [27].
(XLSX)

S4 Table Protein binding analysis among the targets listed in S2 Table. Analysis was performed with the Gene Annotation Tool to Help Explain Relationships GATHER, http://gather.genome.duke.edu/, accessed 05/06/2014 [27].
(XLSX)

S5 Table List of targets obtained by using a cutoff of 3/24 paired samples showing an absolute delta beta between stimulated and baseline avg.beta value of at least 0.1 among the rhGH short-term study cohort. Delta beta values for each sample pair as well as classifiers (“1” for delta beta values >0.1, “0” for delta beta values between 0.1 and -0.1 and “-1” for delta beta values <-0.1) are given.
(XLSX)

S6 Table Gene ontology analysis among the targets listed in S5 Table. Analysis was performed with the Gene Annotation Tool to Help Explain Relationships GATHER, http://gather.genome.duke.edu/, accessed 05/06/2014 [27].
(XLSX)

S7 Table Protein binding analysis among the targets listed in S5 Table. Analysis was performed with the Gene Annotation Tool to Help Explain Relationships GATHER, http://gather.genome.duke.edu/, accessed 05/06/2014 [27].
(XLSX)

S8 Table List of genes overlapping between both approaches (overlap S2 Table and S5 Table). (XLSX)

S9 Table Gene ontology analysis among the targets listed in S8 Table. Analysis was performed with the Gene Annotation Tool to Help Explain Relationships GATHER, http://gather.genome.duke.edu/, accessed 05/06/2014 [27].
(XLSX)

S10 Table Protein binding analysis among the targets listed in S8 Table. Analysis was performed with the Gene Annotation Tool to Help Explain Relationships GATHER, http://gather.genome.duke.edu/, accessed 05/06/2014 [27].
(XLSX)
