# Supplementary figures and images for: In vivo Investigations of the Effect of Short- and Long-Term Recombinant Growth Hormone Treatment on DNA-Methylation in Humans
Source: PLoS One. 2015 Mar 18;10(3):e0120463. doi: 10.1371/journal.pone.0120463 (PMC4364725; doi:10.1371/journal.pone.0120463)

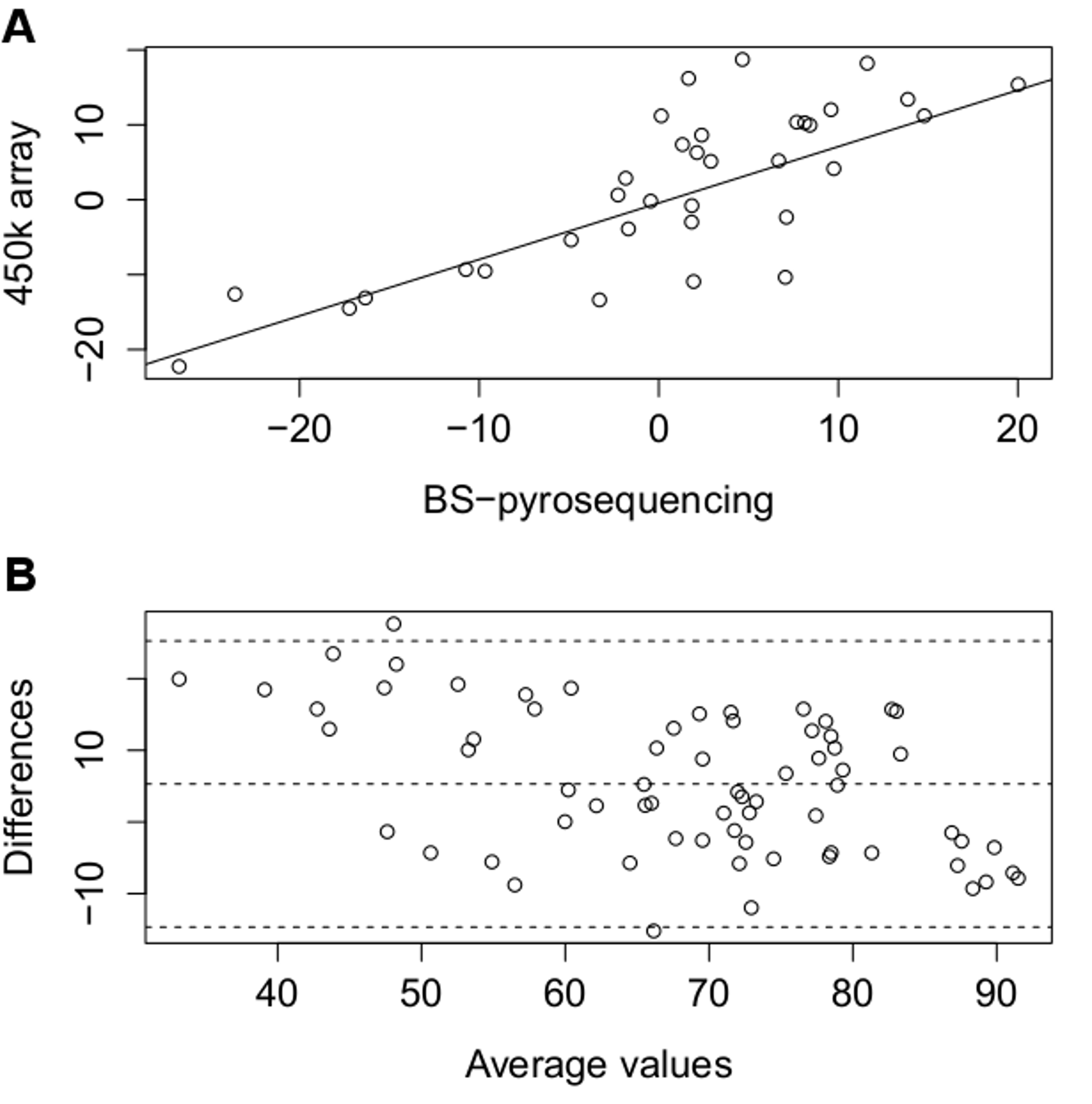

Supplement: S1 Fig — Bisulfite pyrosequencing assays were designed for 5 loci (GNLY, TRIM39, SLC15A4, IGF1R, SLC6A16). Within each locus 2 CpGs covered by the array-based approach were evaluated. For each of the 10 investigated CpGs 1 to 6 sample pairs were analysed, resulting in a total of 34 paired measurements. A: Correlation analysis. Depicted are the delta beta values (value of the stimulated samples subtracted from the value of the baseline sample) for the 34 paired measurements. A comparison of the results obtained by both techniques at the investigated CpGs showed high correlation (Pearson r = 0.78, p-value = 4.015 x 10^-8^). B: Bland-Altman plot. Bland-Altman plot of DNA-methylation results from all 68 single measurements obtained by bisulfite pyrosequencing and the corresponding avg.beta values obtained from the array-based measurements. For this figure avg.beta-values as well as methylation levels determined by bisulfite pyrosequencing are displayed as percent values in order to obtain data sets of the same size range. The difference between both methods for every individual measurement is plotted against the average of both methods. The average of the differences +/− two times the standard deviation denotes the 95% range for the limits of agreement (marked by the dotted horizontal lines). (TIF) [file pone.0120463.s001.tif]
